# Irreversible Electroporation For Liver Tumors: A Review Of Literature

**DOI:** 10.7759/cureus.4994

**Published:** 2019-06-25

**Authors:** Asim Tameez Ud Din, Ahsan Tameez-ud-din, Farooq Mohyud Din Chaudhary, Noman A Chaudhary, Khaleeq H Siddiqui

**Affiliations:** 1 Internal Medicine, Rawalpindi Medical University, Rawalpindi, PAK; 2 Gastroenterology, Nishtar Medical University & Hospital, Multan, PAK; 3 Surgery, Rawalpindi Medical University, Rawalpindi, PAK; 4 Internal Medicine, NewYork-Presbyterian Queens, Flushing, USA

**Keywords:** irreversible electroporation, liver tumors, radiofrequency ablation, microwave ablation, child pugh score

## Abstract

The prevalence of liver tumors is increasing worldwide. These can be broadly classified into primary and secondary types, depending upon the origin of the tumor. Multiple modalities are available for the management of these tumors. Ablative techniques are becoming the cornerstone of management especially for the tumors which are unresectable. Thermal ablative techniques include radiofrequency ablation (RFA), microwave ablation (MWA), and cryotherapy. Recently, a non-thermal technique known as irreversible electroporation (IRE) is gaining importance owing to its better clinical outcome and a good safety profile. IRE works by high voltage and intensity electrical discharge which makes pores in the membrane of the cells. Its clinical outcome is reported in different studies in terms of progression-free survival (PFS), frequency of complete ablation, and local recurrence of the tumor. Favorable results were seen especially for the small size tumors and very early hepatocellular carcinoma (HCC). It was also found to be useful for the management of tumors which are close to vital structures of the liver. The adverse effects of IRE are also comparable to other ablative techniques like RFA and MWA. The common complications associated with this procedure include liver abscess, bleeding, renal failure, pleural effusion, fever, and partial portal vein thrombosis. In view of this literature review, IRE is found to be a good alternative for the management of liver tumor in patients who cannot undergo surgery, thermal ablative procedures or tumor lying close to vital structures. The safety profile of this procedure is also encouraging. Further studies and clinical trials need to be done to explore this technique.

## Introduction and background

Hepatic tumors are one of the leading causes of cancer-related mortality worldwide. In 2018, 841,080 new cases of liver cancers were found all around the world and 781,631 deaths were also reported during this time [1]. Hepatic cancers can be broadly classified into two types, primary and secondary. Primary tumors mainly include tumors arising from the liver or its surrounding structures like the bile duct. It mainly comprises of hepatocellular carcinoma (HCC) and cholangiocarcinoma. Secondary tumors are those which are metastasized to the liver from different parts of the body. The most common sites include the breast, lungs, colon, and rectum. There are different etiologies of these cancers. One of the most common etiology of HCC is liver cirrhosis which is mainly caused by hepatitis, alcohol and non-alcoholic steatohepatitis (NASH) [2]. Multiple treatment options are available for the treatment of hepatic tumors and it is mainly dictated by the stage of the tumor, its location and general health of the patient. Initially, liver resection was the only therapy available but there are a lot of recent advancements in ablative techniques which have changed the conventional treatment of hepatic tumors. There are two types of ablative techniques available. The thermal ablative methods include radiofrequency ablation (RFA), microwave ablation (MWA) and cryotherapy. Recently, a non-thermal technique called irreversible electroporation (IRE) is gaining popularity for the treatment of liver tumors. It works by generating high voltage electrical impulses which render membrane of the cells permanently porous. Due to this non-thermal mechanism of action, this technique can be very useful for the tumors which are inoperable or are located near the vital structures where surgery or thermal ablation is not possible [3]. Our goal was to do the literature review of IRE regarding its efficacy, safety, and comparison with other ablative techniques.

## Review

Methods

A literature search was performed using MeSH terms and keywords on the PubMed and Cochrane library databases. Search results were not confined to a specific geographical area. A total of 55 articles were identified. Original studies demonstrating the efficacy, clinical outcomes, safety profile, and comparison of IRE with other ablation techniques in patients with HCC or secondary liver tumors were included.

The titles and abstracts were initially screened by two independent reviewers. For the screening of full text of articles, a similar protocol was used. Any conflicts were solved with thorough discussion. After doing a detailed discussion and screening, 15 studies were included.

The data was extracted by one author which was reviewed again by a second author. The following variables were analyzed: author, year, study design, sample size, stage of HCC, adjunct therapy, age, cause of HCC/previous history of liver disease, tumor size, follow-up, complications, minor adverse effects, previous treatment, Child-Pugh score, efficacy, clinical response and deaths.

Efficacy and clinical outcomes

There are multiple studies evaluating the efficacy of IRE in the management of liver tumors, both primary and secondary cancers. Kalra et al. conducted a retrospective study in which 21 patients with HCC were treated with IRE. 17 patients had a diagnosis of stage A and four had stage C cancer according to the Barcelona clinic for liver cancer (BCLC) staging system. Child-Pugh score of class A and class B was present in 16 and five patients, respectively. The median size of the tumor as described in the study was 26 mm. At the follow-up of one-month, complete ablation of the tumor was reported in all patients which was defined as an absence of any observed “arterial hypervascularity or wash out in portal venous or delayed phase of CT scan”. Recurrence in the local region was found in five patients (24%). Median progression-free survival (PFS) for the local tumor was seven months and it was mainly affected by tumor size [4].

In another study, Sutter et al. performed a retrospective case series of 58 patients in which treatment of 75 HCC nodules was done through IRE. The median size of the tumor was 24 mm. Child-Pugh score of A and B was present in 46 and 12 patients respectively. Encouraging results were shown in terms of complete ablation of the tumor. Out of 75 tumors, 69 (92%) were completely ablated after three IRE procedures. PFS for local tumor observed at six and 12 months of follow-up was 87% (95% confidence interval [CI] = 77%, 93%) and 70% (95% CI = 56%, 81%) respectively [3]. A prospective study done by Sugimoto et al. in Japan included five patients with six HCC lesions and mean size of 17.5 mm. Out of six, only one tumor showed local recurrence detected by gadoxetic acid-enhanced magnetic resonance imaging (MRI) [5].

Zeng et al. performed a prospective study to assess the efficacy of IRE in large liver tumors (mean diameter 7.2± 2.2 cm) as compared to medium tumors (mean diameter 3.5 ± 0.5 cm). Out of eight patients in the large group, only two showed complete ablation (25%) while 75% had residual disease. In the medium group, better outcomes were reported. Four out of six patients showed complete ablation (66.6%) and 33.3% had a residual disease [6]. A non-randomized clinical trial conducted by Fruhling et al. involved 30 patients with 38 liver tumors which were treated by IRE. Among these tumors, 23 were colorectal cancer which had metastasized to the liver, eight HCC and seven were other primary tumors metastasized to the liver. Median tumor size was 24 mm. At the three- and six-months follow-up, local recurrence was reported in 21.1% and 34.2% patients respectively [7].

A retrospective study conducted by Niessen et al. enrolled 71 patients with 103 tumors of the liver treated by IRE and a median short axis diameter of 1.9 cm. Complete ablation was observed in 92.2% (95/103) of the tumors at six weeks of follow-up. But after a median follow-up of 35.7 months, recurrence of the tumor occurred in 31.7% (33/103) of the treated tumors [8].

Another prospective study was done by Niessen et al. in 34 patients with 65 metastasized liver tumors and median tumor size of 2.4 ± 1.4 cm. In three tumors, retreatment was required due to incomplete ablation on a follow-up period of six weeks. Local recurrence documented at three- and six-month period in nine tumors was the other reason for retreatment. Local recurrence-free survival (LRFS) demonstrated at three, six- and 12 months follow-up was 87.4%,79.8%, and 74.8% respectively [9]. Cannon et al. studied the efficacy of IRE treatment in 44 patients with liver tumors. These tumors were present in close vicinity of important structures of the liver. LRFS reported on three, six and 12 months of follow-up was 97.4%, 94.6%, and 59.5%. respectively. For tumors <3 cm, LRFS at 12 months was reported to be 98% [10].

A recent study was conducted by Mafeld et al. to assess the efficacy of IRE in 52 patients with 59 tumors comprising of primary as well as secondary liver tumors. Complete ablation was observed in 75% (44/59) of the tumors. The survival of patients was reported to be 90% (95% CI 72-97%), 65% (95% CI 40-81%), 52% (95% CI 22-75%) at 12, 24 and 36 months respectively. The median time of progression was found to be eight months but was significantly faster in the colorectal cancer metastatic group than the HCC group [11]. Cheung et al. studied the efficacy of IRE in 11 patients with 18 HCC tumors. The mean diameter of the lesions was 2.44± 0.99 cm. 13 out of 18 lesions (72%) demonstrated complete response “which was defined as no residual or recurrent disease at or directly adjacent to the treated location after up to two ablation treatments and at least six months of follow-up” [12]. Beyer et al. studied the differences between manual IRE method and robot assistance IRE. Robotic assistance IRE was found to be superior owing to less exposure to radiation and high accuracy [13].

To summarize these studies, four main predictors of efficacy and clinical outcomes of IRE are highlighted. The first one is the complete ablation of tumors. In a recent study by Karla et al., complete ablation was reported in all patients after a follow-up of one month [4]. This was in contrast to other studies. Niessen et al. and Mafeld et al. demonstrated complete ablation in 92.2% and 75% of the tumors at six weeks and four to eight weeks of follow-up respectively [8,11]. Zeng et al. compared IRE efficacy in large and medium-size liver tumors. Complete ablation was higher in the medium group (66.6% of the patients) than the large group (25% of the patients) [6]. This shows the higher efficacy of IRE in the management of small to medium-sized tumors as compared to large tumors. The second predictor is tumor recurrence. Local recurrence was observed in five patients (24%) in Karla et al. study [4]. In other studies, like Fruhling et al. and Niessen et al., it was observed in 21.1% and 31.7% of the patients, at three months and 35.7 months of follow-up respectively [7-8]. The third predictor is LRFS. At three, six- and 12-months follow-up, LRFS reported in Niessen et al. and Cannon et al. studies were 87.4%,79.8%, 74.8% and 97.4%, 94.6%, 59.5% respectively [9-10]. In Cannon et al., for small sized tumors (<3 cm) LRFS was increased to 100%,100% and 98% at three, six and 12- months follow up [10]. The fourth predictor discussed is PFS. In Karla et al. study, the median PFS for the local tumor was found to be seven months [4]. The median time for local tumor progression demonstrated in Sutter et al. study was nine months [3]. So, in conclusion, IRE is found to be efficient for the management for liver tumors which are small in size as compared to large tumors. 

Safety profile

Dollinger et al. studied the common adverse events encountered during the IRE procedure. It was a retrospective study of 85 IRE procedures. No mortality was reported. The most common event was a liver abscess in 4.7% (4/85) of the cases. Other complications included hemorrhage which was self-limiting in 5.9% (5/85) of the cases but required transfusion of blood or arterial embolization in one patient each, kidney failure in one patient, pneumothorax which did not require chest intubation in 3.5% (3/85) and liver arteriovenous shunt also occurred in 3.5% (3/85) of the cases. Approximately 2.3% (2/85) of the cases developed some neurologic issues in the upper limb on the right side of the body but patients completely recovered at the time of discharge [14]. 

Other adverse effects highlighted in other studies include fever, mild right pleural effusion, mild hemoperitoneum, subcapsular hematomas, atrial fibrillation and partial portal vein thrombosis [4,11,15].

Comparison with other modalities

Bhutiani et al. compared the efficacy of IRE with MWA for the treatment of HCC with a median tumor size of 3.2 cm. Fifty-five patients were included in the study. Thirty underwent IRE and 25 MWA. Both modalities demonstrated similar success rates at 90 days of treatment (100%). The treatment success rate at 180 days was 97% and 100% in IRE and MWA respectively but it was not statistically significant (p = 0.37). One benefit of IRE over MWA was found in terms of readmittance within 90 days of treatment. Four patients in IRE as compared to nine patients in the MWA group were needed to be readmitted (p = 0.03) [15].

Narayanan et al. compared IRE with RFA technique in terms of pain experienced by the patients after treatment of HCC with either of these procedures. No significant difference was found between the two groups in the mean pain score or doses of opioid (hydromorphone) used for pain management [16]. Verloh et al. compared the incidence of different complications ranging from mild to life-threatening, between thermal ablation (RFA, MWA) and IRE which is a non-thermal ablation technique. Approximately 26.5% (31/117) in RFA/MWA versus 34% (16/47) in patients who underwent IRE showed grade I and II adverse events. Major complication rates were also comparable between the two groups; 2.6% (3/117) versus 2.1% (1/47) in RFA/MWA and IRE groups respectively. So, this study revealed that there is no significant difference in the incidence of complications among these techniques [17].

These studies show that IRE when compared to other ablative techniques, has similar efficacy and complication rates.

## Conclusions

Irreversible electroporation is a relatively new non-thermal ablation technique. It is found to be really useful for the liver tumors which are either not accessible to surgery or there is any contraindication to surgery. It can also be used as an alternative in cases where thermal ablation techniques are not advisable. In the literature review, it is found that IRE is more useful for small liver tumors and very early stage HCC. The complications associated with this procedure are comparable to other ablation techniques. Further studies need to be done to get more insight regarding the efficacy and safety of this procedure.
